# Alternative splicing of EZH2 regulated by SNRPB mediates hepatocellular carcinoma progression via BMP2 signaling pathway

**DOI:** 10.1016/j.isci.2024.111626

**Published:** 2024-12-18

**Authors:** Xingyu Wang, Weiyi Liu, Chunai Zhan, Yuanyuan Zhang, Xinyu Li, Yaoyun Wang, Mengfei Sheng, Madiha Maqsood, Hang Shen, Anmin Liang, Wei Shao

**Affiliations:** 1 Department of Microbiology and Parasitology, Anhui Provincial Laboratory of Pathogen Biology, School of Basic Medical Sciences, Anhui Medical University, Hefei, Anhui 230032, China; 2 Department of Hepatopancreatobiliary Surgery, the First Affiliated Hospital, Anhui Medical University, Hefei 230000, China; 3 College of Life Science, Wuhan University, Wuhan, Hubei 430072, China

**Keywords:** Biological sciences, Molecular biology, Molecular mechanism of gene regulation, Cancer

## Abstract

Increasing evidence suggests that aberrant alternative splicing plays crucial roles in tumorigenesis. However, the function of EZH2 splice variants as well as the mechanism by which EZH2 alternative splicing occurs in hepatocellular carcinoma (HCC) remain elusive. Here, we analyzed both our own and published transcriptomic data, obtaining 19 splice variants of EZH2 in addition to canonical full-length EZH2-A in HCC. We found that expression of EZH2-A/EZH2-B in tumor tissues and cell lines was significantly higher than in normal tissues. Conversely, EZH2-C expression was lower in tumor tissues and cell lines than in normal tissues. Further functional analysis indicated that unlike full-length EZH2-A that promotes H3K27 methylation, EZH2-C reduced H3K27me3 levels. EZH2-C inhibited proliferation, migration, invasion of HCC cells. Moreover, EZH2-A and EZH2-C regulate the BMP2 signaling pathway oppositely. Mechanistically, EZH2’s alternative splicing was mediated by splicing factor SNRPB. In summary, this study revealed that alternative splicing of EZH2 regulates HCC.

## Highlights

• Alternative splicing of EZH2 was analyzed in HCC
• Splice variant EZH2-C functions opposite to EZH2-A and EZH2-B
• SNRPB regulates EZH2 alternative splicing in HCC

## Graphical abstract

**Figure:**
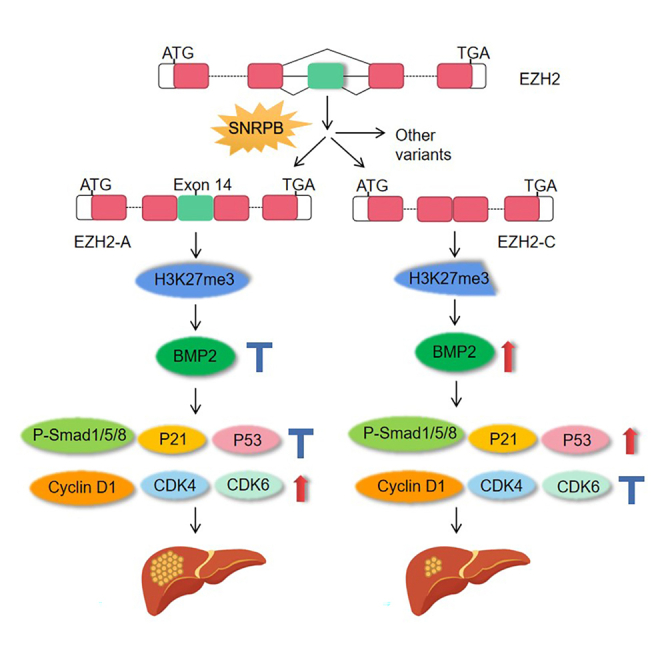

## Introduction

In eukaryotes, pre-messenger RNA (pre-mRNA) splicing is a critical step in gene expression.^1^^,^^2^ Alternative splicing is the process where pre-mRNA undergoes splicing by selecting different splice sites, resulting in multiple types of mRNA.^3^ It is estimated that more than 95% of human genes undergo alternative splicing, which is a pivotal mechanism for generating protein diversity.^4^ Importantly, alternative splicing is involved in almost all aspects of tumor biology, including proliferation, differentiation, cell cycle control, metabolism, apoptosis, motility, invasion, and angiogenesis.^5^^,^^6^ Alternative splicing of many oncogenes is closely associated with the development and progression of cancers, such as breast, prostate, cervical, and hepatocellular carcinoma (HCC).^7^^,^^8^^,^^9^^,^^10^ For example, we demonstrated that a splice variant of the regulator of methyltransferase-like 3 (METTL3) inhibits the proliferation, migration, and invasive activity of HCC cells.^11^ GCH1-L and STK39-L, two longer splice variants of GCH1 and STK39, promote the tumorigenic potential of hepatoma cells, whereas their shorter splice variants have no apparent effects.^12^ However, the detailed mechanism of alternative splicing regulated in HCC remains largely unexplored. Therefore, elucidating the molecular mechanisms of alternative splicing and HCC progression can help to develop effective metastasis-targeted therapies and improve the overall prognosis of patients with HCC.

Liver cancer remains a global health challenge with an estimated incidence of more than 1 million cases by 2025. HCC is the most common form of liver cancer, accounting for 90% of cases. There is an urgent unmet clinical need for preventing, diagnosing, prognosis, and treating this deadly cancer since HCC is one of the most common and aggressive human malignancies in the world.^13^^,^^14^ An increasing number of studies have shown that the development of HCC is closely related to the alterations of alternative splicing.^11^^,^^15^^,^^16^^,^^17^ For example, splicing factor small nuclear ribonucleoprotein polypeptides B and B1 (SNRPB) is highly expressed in many cancers,^18^^,^^19^ including HCC.^16^ Although SNRPB is a core component of the spliceosome, it also plays an important role in the regulation of alternative splicing. A recent study reported that SNRPB were upregulated in HCC, resulting in the formation of AKT3-204 and LDHA-220 splice variants in HCC.^16^ Therefore, aberrant alternative splicing could play a critical role in HCC development and progression. However, the function of differently spliced variants and splicing regulators in HCC need further investigation.

Enhancer of zeste homolog 2 (EZH2) is a member of the polycomb group (PcG) protein family. EZH2 is a histone methyltransferase that catalyzes histone H3 lysine 27 trimethylation (H3K27me3) by the polycomb repressor complex 2, which modulates transcription at the epigenetic level by regulating histone- and DNA-methylation.^20^^,^^21^^,^^22^ Numerous studies have shown that transcription of many tumor suppressor genes is repressed by EZH2 in malignant tumors, suggesting that EZH2 dysregulation plays a key role in carcinogenesis. In addition, EZH2 can mediate the promoter hypermethylation-mediated silencing by recruiting DNA methyltransferases to the CpG-rich promoter regions of target genes. EZH2 is highly expressed in several cancer types and has been associated with progression and metastasis in HCC, bladder cancer and breast cancer.^23^^,^^24^ As EZH2 acts as an epigenetic silencer via its function of regulating H3K27me3, it plays an important role in tumorigenesis and tumor progression.^25^^,^^26^ Alternative splicing of EZH2 was found to contribute to tumorigenesis.^27^^,^^28^ However, the detail function and mechanism of EZH2 splicing in HCC are still largely unexplored.

In this study, alternative splicing of EZH2 was analyzed in normal liver and HCC tissues. We found that the EZH2-C variant functions differently compared with the canonical full-length EZH2-A in HCC. EZH2-C was significantly associated with early HCC tumor stages and longer overall survival times. EZH2-A and EZH2-C oppositely controlled the expression of EZH2 downstream through the BMP2 signaling pathway. In addition, silencing of the splicing factor SNRPB promotes the splicing switch from EZH2-A to EZH2-C, thereby affecting the expression of EZH2 regulated genes.

## Results

### Identification of EZH2 alternatively spliced variants

**Figure 1:**
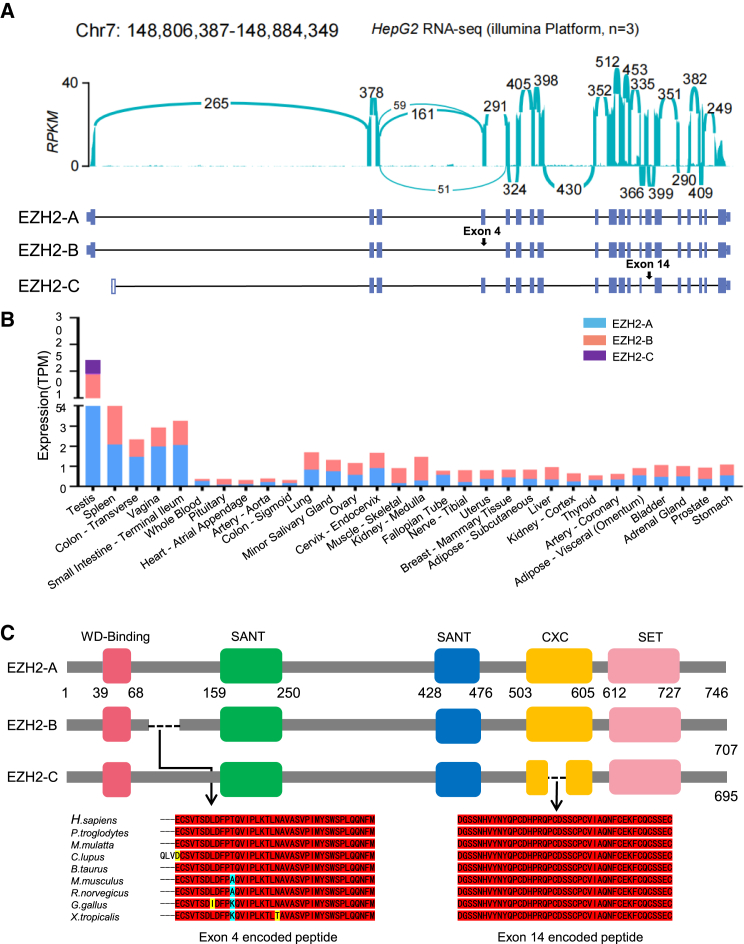
Identification of alternative splicing variants of EZH2 (A) Analyses of alternative splicing of EZH2 using online and our transcriptomic data. Upper, Sashimi plot of EZH2 using the aligned RNA-seq reads from HepG2 cells. Lower, comparison of the three EZH2 splice variants. (B) mRNA levels of EZH2-A, B and C in human tissues. Analyses were performed using data from GTEx Portal. (C) Comparison of protein domains of the EZH2 splice variants. Peptide sequences encoded by exon 4 and exon 14 of EZH2 from *Homo sapiens, Pan troglodytes, Macaca mulatta, Canis lupus, Bos taurus, Mus musculus, Rattus norvegicus, Gallus gallus, and Xenopus tropicalis* are presented.

The human EZH2 gene has 20 canonical exons located on Chr7. To thoroughly examine the splice variants of EZH2 in HCC, we first analyzed our next-generation RNA-seq data of the HCC cell line HepG2. Including the full-length EZH2 canonically spliced product (named EZH2-A here), twenty transcripts from the EZH2 gene in HepG2 cells were detected, all of which can be found in the online Ensembl database (Figure 1A; Figure S1A).

Considering the expression levels of EZH2 transcripts and their lengths, and also based on HepG2 third generation RNA-seq data (Figure S1B), we chose two splice variants (named EZH2-B, -C here) as well as full-length EZH2-A for further study. Expression of EZH2 splice variants in human tissues were analyzed using GTEx Portal. Ten of the twenty isoforms were detected in human tissues with different expressions (Figure S1C). Further analysis showed that EZH2-A, -B, were relatively higher expressed in all the analyzed tissues. EZH2-C was relatively lower expressed in tissues (Figure 1B). The full-length EZH2-A has 20 canonical exons and translates into a protein of 746-aa. In the variant EZH2-B, exon 4 is skipped, producing a 707-aa protein without a part of the peptide between the WD-Binding domain and the SANT domain. In the EZH2-C variant, exon 14 is skipped, resulting in 695-aa protein which lacks the central region of the CXC domain (Figure 1C; Figure S2A).

EZH2 has five functional domains: the WD binding domain (WD-Binding) that interacts with EED; N-CoR and TFIIIB (SANT) that interact with histones; a cysteine-rich domain (CXC), CXC domain is required for interactions with other PRC2 components and regulatory proteins; and the suppressor of variegation 3–9, enhancer of zeste and trithorax (SET) catalytic domain, the conserved SET domain at the C-terminus functions for maintaining histone methyl transferase (HMT) activity.^29^^,^^30^ We further analyzed the peptide encoded by EZH2 exon 4 and exon 14, which is skipped in EZH2-B and EZH2-C, respectively. We found that they are highly conserved in vertebrates (Figure 1C; Figure S2A), indicating the EZH2-B and EZH2-C could be functionally important. In addition, we also predicted the protein structures of EZH2-A, B, and C by AlphaFold (https://alphafoldserver.com/) and found that alternative splicing of EZH2 results in dramatic change of EZH2 structures (Figure S2B).

### EZH2-C is negatively associated with HCC malignancy

**Figure 2:**
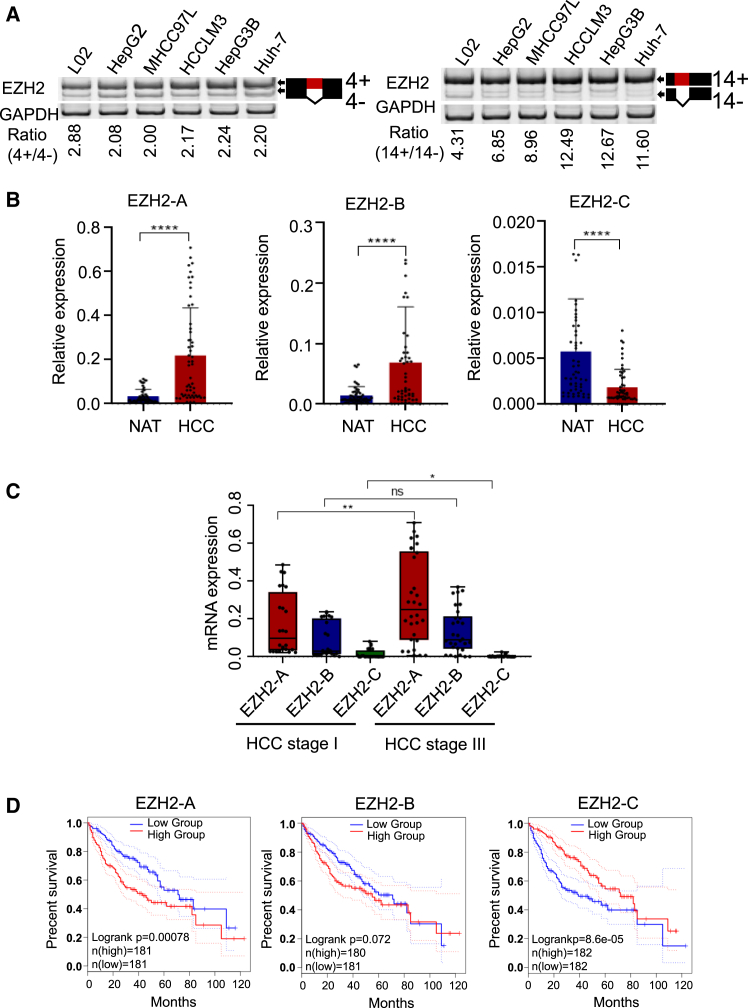
EZH2-C is negatively associated with HCC malignancy (A) Left, expression of EZH2-4+ and EZH2-4- mRNA in a normal hepatocyte line (L02) and five hepatocellular carcinoma cell lines (HepG2, MHCC97L, HCCLM3, HepG3B, Huh-7) measured by RT-qPCR. Ratio for 4+/4- is listed below the panel. Right, expression of EZH2-14+ and EZH2-14- mRNA measured by RT-qPCR. Ratio for 14+/14- is listed below the panel. (B) mRNA levels of EZH2-A, B, and C in NATs and tumor tissues from our patients were measured using RT-qPCR. The clinical characteristics of all the patients are listed in Table S1. NAT: normal tissue adjacent to the tumor. (C) mRNA levels of EZH2-A, B and C are correlated with tumor stage. The mRNA levels were analyzed by RT-qPCR, and patients were grouped as early-stage HCC (Stage I) and late-stage HCC (Stage III). (D) HCC patients with higher levels of EZH2-C had longer overall survival times (TCGA data).Data are presented as means ± SD. ∗*p* < 0.05, ∗∗*p* < 0.01, ∗∗∗*p* < 0.001, ∗∗∗∗*p* < 0.0001 (t test). Abbreviations: HCC, hepatocellular carcinoma.

To explore the function of EZH2 alternatively spliced variants in HCC, we first used RT-PCR to detect the expression of EZH2-A, -B and -C. While there is not much difference was seen between EZH2-A and -B (exon 4 skipping) by the endpoint PCR assay (Figure 2A, left), the PCR signal for the EZH2-C (exon 14 skipping) was clearly decreased in HCC cell lines (Figure 2A, right). RT-qPCR was also performed to quantify the expression of EZH2-A/B/C in normal liver cell line L02 and liver cancer cell lines, which identified that EZH2-A is the most abundant splice variant, but EZH2-B/C was also expressed in the cells (Figure S3A).

We also measured the mRNA expression of EZH2-A/B/C using human (clinical) samples. We found that expression of EZH2-A and -B was significantly increased in the HCC tumor tissues compared with their NATs while EZH2-C was significantly suppressed in tumor tissues (Figure 2B). Western blotting also showed that EZH2-A/B is highly expressed in HCC cell lines (Figure S3B) and human samples (Figure S3C). Also, by analyzing TCGA data, EZH2-A and EZH2-B were found to be upregulated in most solid tumors (Figure S4). However, the signals for the expression of EZH2-C in cancers were below the detection limit in TCGA database and western blotting experiments.

To explore the roles of EZH2 splice variants in HCC, we analyzed correlations between the EZH2-A/B/C expression and the TNM stages of HCC patients. Eighteen patients were classified into two groups according to their developmental stages of tumors (Table S1). EZH2-A was at a relatively higher level in patients in the later tumor-stage than in earlier-stage patients. In contrast, the EZH2-C level in patients with early-stage tumors is higher than that in patients with advanced stages (Figure 2C). Further analyses using survival data from TCGA showed that HCC patients with high levels of EZH2-A and B exhibited shorter overall survival. Also, after normalizing the expression level of EZH2-C to that of the total EZH2, we found patients with high levels of EZH2-C live longer (Figure 2D). Although the differences were not significant, similar survival patterns were also observed in patients with other solid tumors, such as MESO, SARC (Figure S5).

### EZH2-C inhibits the proliferation, migration, invasion and EMT processes of HCC cells

Given the importance of EZH2 in directing tumor growth and metastasis,^31^ we next assessed the tumorigenicity of EZH2 variants. Flag-tagged EZH2 variants were constructed for functional analysis. Western blot showed that the EZH2-A/B/C proteins were stably expressed (Figure S6A). Immunofluorescence assay indicates that these three EZH2 variants are mainly localized in the nucleus (Figure S6B).

**Figure 3:**
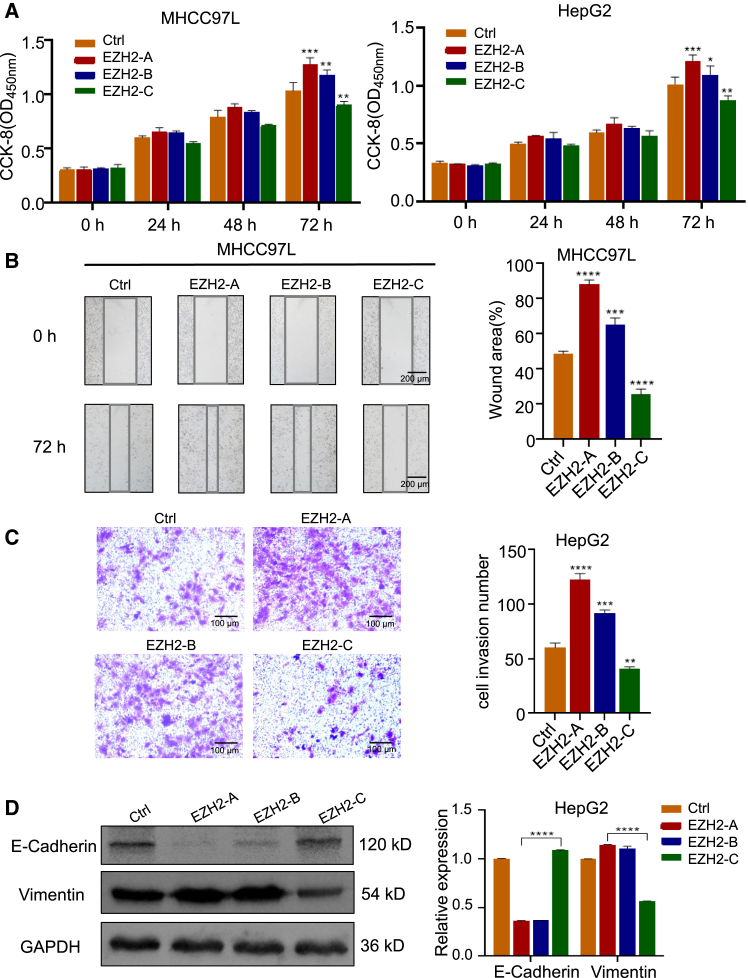
EZH2-C inhibits the proliferation, migration, invasion and EMT processes of HCC cells (A) EZH2-A, -B or -C plasmids were transfected into MHCC97L cells (left), or HepG2 cells (right). The cell proliferative activity was assessed by CCK-8 assay. (B) Expression of EZH2-C inhibits the migration of MHCC97L cells. Representative images are shown at 0 h and 48 h after transfections. Scale bar, 200 μm. (C) Expression of EZH2-C inhibits the invasion ability of HepG2 cells. Scale bar, 100 μm. (D) Western blot analysis of epithelial and mesenchymal markers in HepG2 cells ectopically expressing EZH2-A, -B or -C. GAPDH was used as a loading control. Data are presented as means ± SD. ∗*p* < 0.05, ∗∗*p* < 0.01, ∗∗∗*p* < 0.001, ∗∗∗∗*p* < 0.0001 (t test).

We found that overexpression of canonical full-length EZH2-A promoted the growth and proliferation of MHCC97L and HepG2 cells. EZH2-B was also found to promote the growth and proliferation although the effects were milder. By contrast, overexpression of the EZH2-C variant suppressed cell growth and proliferation (Figure 3A). We further used splice variants specific siRNA to knock down EZH2-A, B and C in MHCC97L (Figure S7A), and found that silencing of EZH2-A and B inhibits HCC cell growth and silencing of EZH2-C promotes HCC cell proliferation (Figure S7B).

Moreover, the wound healing assay showed that the migration ability of HCC cells was enhanced by overexpression of EZH2-A and EZH2-B but inhibited by EZH2-C in both MHCC97L (Figure 3B) and HepG2 (Figure S8A) cells. A similar phenotype was observed in normal liver cell line L02 (Figures S8B and S8C). Moreover, the invasion abilities of the MHCC97L cells were enhanced by overexpression of EZH2-A and EZH2-B but inhibited by EZH2-C (Figure 3C).

We also examined expressions of epithelial-mesenchymal transition (EMT) marker after overexpression of EZH2-A, B and C. Consistently, expressions of Vimentin (mesenchymal marker) were reduced whereas E-cadherin (epithelial marker) was upregulated after EZH2-C overexpression in HepG2 cells (Figure 3D), suggesting that EZH2-C inhibits EMT. These results indicate that, unlike EZH2-A and EZH2-B, the EZH2-C variant could be a tumor suppressor that inhibits proliferation, migration, invasion, and EMT of HCC.

### EZH2 variants have different abilities to catalyze the trimethylation of H3K27

**Figure 4:**
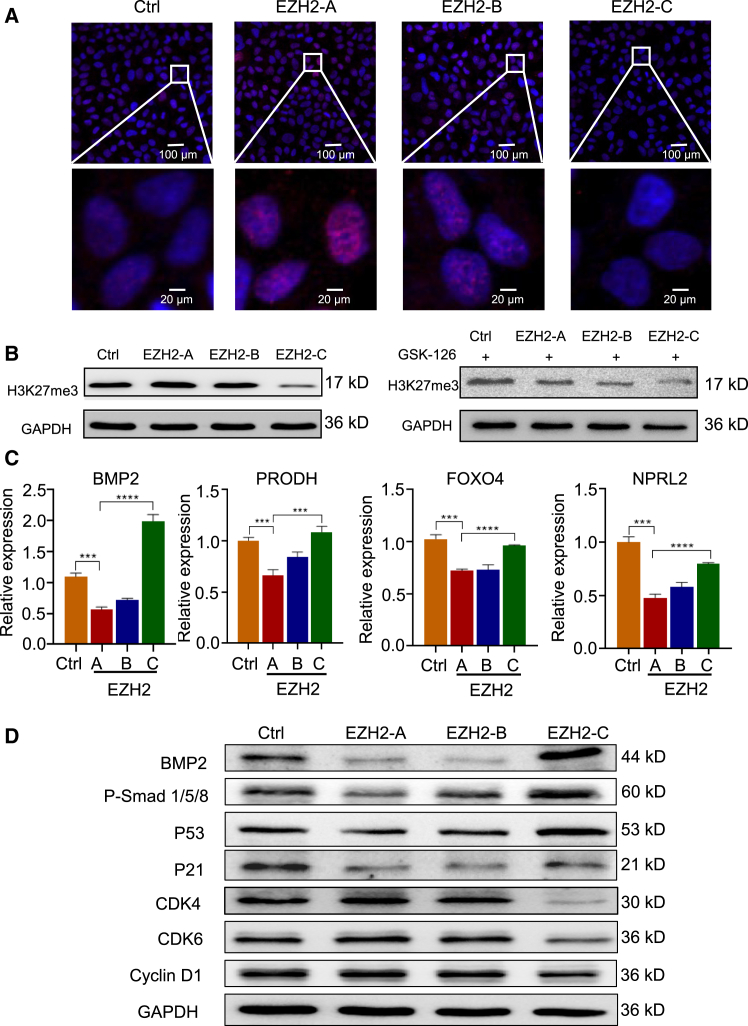
Compared with the canonical full-length EZH2-A, EZH2-C functions oppositely to downstream genes (A) Immunofluorescence analysis of H3K27me3 in HepG2. Scale bar,100 μm (upper), 20 μm (down). (B) Western blot analysis of H3K27me3 expression in cells treated with or without GSK-126. (C) RT-qPCR analyses of the mRNA levels of EZH-2 downstream target genes in HepG2 cells expressing EZH2-A, B or C. (D) Western blot analysis of the proteins involved in BMP2 signaling pathway in HepG2 cells expressing EZH2-A, B or C. GAPDH was used as a loading control. Data are presented as means ± SD. ∗*p* < 0.05, ∗∗*p* < 0.01, ∗∗∗*p* < 0.001, ∗∗∗∗*p* < 0.0001 (t test).

EZH2 functions by catalyzes the trimethylation of histone H3 lysine 27 (H3K27me3).^20^^,^^32^ Therefore, we measured the histone methyltransferase activity of the EZH2 variants by immunofluorescence assay in HepG2 cells. The H3K27me3 level was significantly or moderately increased in cells expressing EZH2-A (WT) or EZH2-B, respectively while it was decreased in cells expressing the EZH2-C variant (Figure 4A). Western blotting also indicated that EZH2-C strongly decreased the H3K27me3 level in HepG2 cells while EZH2-B slightly decreased it. After cells were incubated with GSK-126, an inhibitor of EZH2,^33^ the H3K27me3 levels were all decreased (Figure 4B), confirming that the changes in the levels of H3K27me3 were due to the activity of EZH2. There results suggest that EZH2-C has decreased histone methyltransferase activity, which is consistent with bioinformatic analysis that the CXC structural domain is disrupted in EZH2-C (Figure 1C).

### Unlike EZH2-A, EZH2-C promotes BMP2 signaling pathway in HCC

Increasing numbers of studies have demonstrated that EZH2 participates in many diverse biological processes and control the expression of many downstream genes in different cancers.^20^^,^^22^^,^^34^^,^^35^ EZH2 catalyzes tri-methylation of H3K27 to regulate gene expression through epigenetic machinery.^36^ To investigate the function of EZH2 variants on downstream gene expression, we performed RT-qPCR analysis of known EZH2 downstream genes in HepG2 cells. We found that both EZH2-A and -B subtypes inhibited the expression of these four downstream cancer related genes (BMP2, PRODH, FOXO4, NPRL2), while EZH2-C exhibited the opposite effect (Figure 4C). Moreover, splice variants specific knocking down of EZH2-A, B and C showed opposite four genes’ expression compared with overexpression of EZH2 variants (Figure S9).

We found that Bone Morphogenetic Protein 2 (BMP2) was highly induced by EZH2-C. Therefore, we investigated the effect of EZH2 variants on the BPM2 signaling pathway. BMP2 belongs to the transforming growth factor β (TGF-β) superfamily. Aberrant expression of BMP2 has been reported in several cancer tissues.^37^ In the BPM2 signaling pathway, BMP2 reduces the expression of cyclin D1, inhibits cyclin-dependent kinases CDK4 and CDK 6 activities, and stimulated *p*-Smad1/5/8, p53, and p21 levels.^38^ In our study, we found that consistent with RT-qPCR results, EZH2-C induced BMP2 expression while EZH2-A and -B greatly reduced its expression. We also identified that expression of *p*-Smad1/5/8, p53, and p21 was increased while that of cyclin D1, cyclin-dependent kinase 4 (CDK4), and CDK6 was decreased in cells expressing EZH2-C while EZH2-A and -B greatly showed the opposite effect (Figure 4D). These results indicate that EZH2 alternative splicing could regulate HCC via BPM2 signaling pathway.

### SNRPB regulates EZH2 alternative splicing in HCC

Mis-splicing in cancers can be regulated by different splicing factors.^3^^,^^39^^,^^40^ Previous studies have shown that SF3B3 regulates EZH2 splicing in renal cancer and HCC.^27^^,^^28^ However, alternative splicing is a complicated process involved with many regulators.^41^^,^^42^

**Figure 5:**
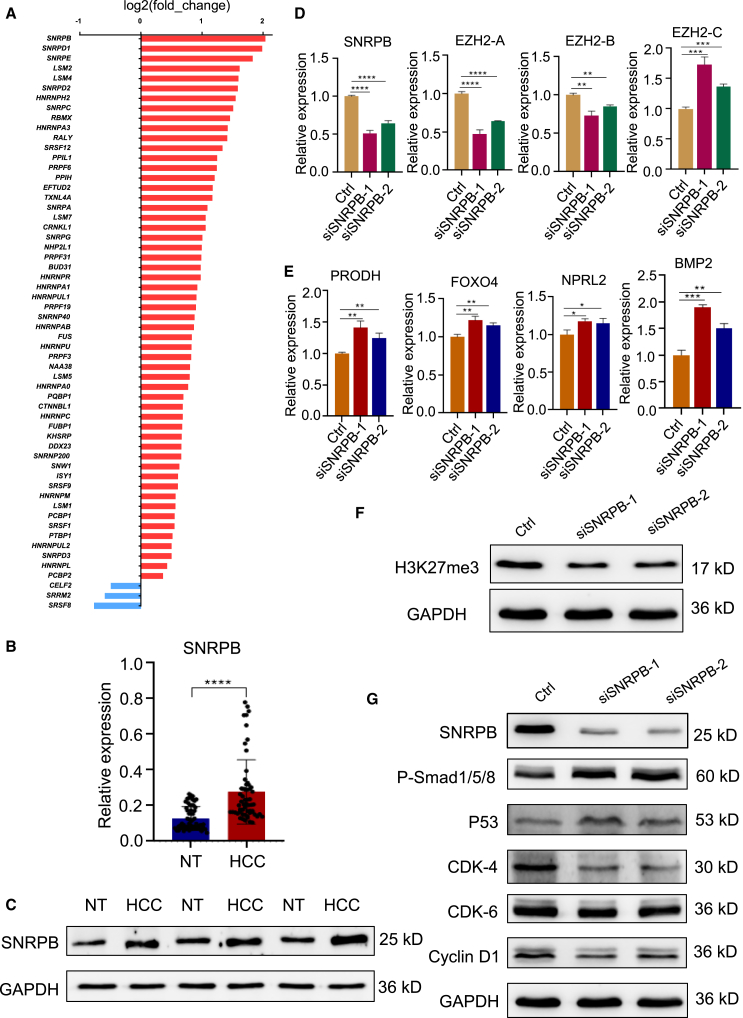
SNRPB regulates alternative splicing of EZH2 in HCC (A) Splicing factors with altered expression in HCC tissues compared to adjacent normal liver tissues based on TCGA database. (B) SNRPB was upregulated in hepatocellular carcinoma. Total RNA isolated from paired HCC tumors and adjacent normal tissues was subjected to RT-qPCR. (C) Western blot analysis of the SNRPB protein in normal and tumor tissues of patients with hepatocellular carcinoma. (D) RT-qPCR analysis of SNRPB and EZH2 isoforms after SNRPB knockdown. (E) RT-qPCR analysis of genes downstream of EZH2 after SNRPB knockdown. (F) Detection of H3K27me3 after SNRPB knockdown by western blotting. (G) SNRPB knockdown suppressed the BMP2 signaling pathway. Data are presented as means ± SD. ∗*p* < 0.05, ∗∗*p* < 0.01, ∗∗∗*p* < 0.001, ∗∗∗∗*p* < 0.0001 (t test).

To explore the splicing factor that regulates EZH2 alternative splicing in HCC, we analyzed published data containing 50 paired HCC samples (GSE77314) and identified a set of splicing factors that significantly increased or decreased in HCC tissues. Among them, splicing factor SNRPB was identified to be highly expressed and significantly induced in HCC (Figure 5A). We then confirmed the higher/induced expression of SNRPB by RT-qPCR analysis of 20 paired HCC and non-tumor liver tissues (Figure 5B). Moreover, three paired liver cancer samples were analyzed by western blotting, and the results were consistent with the RT-qPCR results (Figure 5C). To investigate whether SNRPB regulates EZH2 alternative splicing, we knocked down SNRPB by siRNA (Figure 5D, left). Interestingly, EZH2-A/B expression was decreased while EZH2-C expression was increased after silencing of SNRPB (Figure 5D, middle and right). These results indicate that SNRPB regulates EZH2 alternative splicing in HCC cells. We also found that EZH2 downstream genes were also increased (Figure 5E), and the H3K27me3 level was decreased after silencing of SNRPB (Figure 5F).

Next, we investigated whether SNRPB could also regulate the BPM2 signaling pathway. We found that SNRPB knockdown decreased the expression of cyclin D1, CDK4, CDK6 expression, and increased expression of *p*-Smad1/5/8 and p53 (Figure 5G). These results suggest that SNRPB could be a potent regulator of EZH2 alternative splicing in HCC.

## Discussion

Alternative splicing is a key mechanism for expanding the expression diversity of mammalian genes.^43^^,^^44^ Alternative splicing of oncogenes produces splice variants that are associated with the progression of many cancers, including HCC.^45^^,^^46^^,^^47^ EZH2 is a core member of the PRC2 protein family and a functional enzyme component of PRC2, EZH2 acts as a histone methyltransferase that influences the development of a variety of cancers.^31^^,^^48^^,^^49^ EZH2 is upregulated in tumor tissues of HCC patients, influencing transcription at the epigenetic level and promoting the progression of HCC.^20^^,^^21^^,^^22^ In this study, we identified a splice variant of EZH2, EZH2-C, which has a function opposite to the canonical full-length EZH2 (named here as EZH2-A) based on three line of evidence; (1) Expression analysis of mRNAs showed that the levels of EZH2-A in normal liver tissues were lower than those in NAT of liver cancer patients while the levels of EZH2-C in normal liver tissues were higher than those in NAT of liver cancer patients. (2) In contrast to EZH2-A that promotes H3K27me3 methylation and mRNA decay of downstream target genes, EZH2-C inhibits H3K27me3 methylation and increases mRNA stability of downstream genes. (3) EZH2-C is negatively correlated with the malignancy of HCC, determined by the cell growth, migration and invasion whereas it is positively correlated with longer survival of patients. Therefore, we propose a novel mechanism of oncogene inactivation in HCC that regulates histone and DNA methylation at the epigenetic level to affect transcription, where the selective splice variants of EZH2 antagonize the function of its full-length isoform and repress H3K27me3 methylation, thereby altering the expression of the oncogene.

EZH2-A, B and C are functionally important in the progression of HCC. Other splicing variants of EZH2 either have low expression levels or harbor a premature termination codon (PTC) which commits the splicing variants to degradation. EZH2-C, which disrupted the CXC domain highly reduces the methyltransferase activity but didn’t abolish the activity. This finding supports the notion that the CXC structural domain of EZH2 is required for histone methyltransferase activity.^50^ We speculate that EZH2-C acts mainly by counteracting full-length EZH2. A previous study reported that a splice isoform similar to EZH2-C was also found in kidney cancer, where EZH2-C also has an opposite role to EZH2-A.^27^ EZH2-C is expressed more in normal tissues than in tumor tissues. Furthermore, overexpression of EZH2-C in HCC cells inhibits biological processes such as tumor cell proliferation, invasive migration, and invasion. These results suggest that the truncated EZH2-C protein is a key regulator of tissue homeostasis. Aberrant splicing which reduces EZH2-C level plays an important role in HCC progression.

Aberrant regulation of alternative splicing is a very important molecular feature of cancer,^11^^,^^51^ yet the detailed mechanisms and biological consequences remain elusive. Previous studies have demonstrated that splicing factors play a key role in splice site recognition and assembly of spliceosomes during alternative splicing. Splicing factor SNRPB has been shown to mediate RNA splicing to promote HCC cell proliferation.^16^ In our study, SNRPB expression was elevated in HCC tissues, and SNRPB knockdown promoted the shift from EZH2-A splice isoform to EZH2-C isoform, decreased histone methyltransferase activity, reduced H3K27 trimethylation, and led to impaired expression of cell cycle-related proteins, thereby inhibiting HCC cell survival via BMP2 signaling pathway. In conclusion, our results represent a novel mechanism of how SNRPB is involved in HCC cell survival through the regulation of cancer-related genes.

In summary, our study suggests that SNRPB mediates the alternative splicing of EZH2. Patients with HCC with high EZH2-C levels and low EZH2-A levels have longer overall survival, thus EZH2-C and EZH2-A could be used as prognostic markers for postoperative survival in HCC. Moreover, the production of the new splice variant EZH2-C could inhibit the growth of HCC cells, thus aberrant EZH2 splicing plays a key role in tumorigenesis. In the future, SNRPB-mediated splicing switch of EZH2-A to EZH2-C may be a potential therapeutic approach for HCC. In addition, elucidating the molecular mechanisms of HCC progression could help develop targeted therapies and improve the overall prognosis of patients.

### Limitations of the study

This study has been focusing on the canonical function of EZH2 in H3K27 methylation. However, EZH2 also has many non-canonical functions. Future work exploring the non-canonical functions of EZH2 variants will fully elucidate their roles in HCC. Our analysis investigated the function of three major EZH2 splice variants in HCC. The function of other EZH2 splice variants is unknown. Alternative splicing of EZH2 is not fully understood. Future work using third-generation sequencing combined with second generation sequencing in different samples could identify more EZH2 splice variants.

## Resource availability

### Lead contact

Further information and requests for resources and reagents should be directed to and will be fulfilled by the lead contact, Wei Shao (ShaoWei@ahmu.edu.cn).

### Materials availability

All unique/stable reagents in this study can be generated using protocols detailed in the STAR Methods section or can be requested from the lead contact.

### Data and code availability

• HepG2 transcriptome sequencing data have been deposited at GEO and are publicly available as of the date of publication. Accession numbers are listed in the key resources table.
• This paper does not report original code.
• Any additional information required to reanalyze the data reported in this paper is available from the lead contact upon request.

## Acknowledgments

The authors thanks Koh Fujinaga (University of California, San Francisco) for proofreading the manuscript. The authors also acknowledge The ENCODE Project Consortium and Ali Mortazavi laboratories for making their third-generation sequencing data publicly available.This work was supported by the 10.13039/501100001809National Natural Science Foundation of China (82071770); Research Level Improvement Project of Anhui Medical University (2021xkjT001); 10.13039/501100003995Anhui Provincial Natural Science Foundation (2008085QH371); Scientific Research of BSKY in Anhui Medical University (XJ201601); Research and practical innovation projects of AHMU (YJS20230039).

## Author contributions

W.S. designed the theme and direction of the manuscript. X.W., W.L., C.Z., Y.Z., X.L., Y.W., M.S., M.M., and A.L. performed all the experiments. H.S. collected HCC samples. X.W., M.S., and W.S. wrote, reviewed, and edited the manuscript.

## Declaration of interests

The authors declare no competing interests.

## STAR★Methods

### Key resources table

**Table:** 

REAGENT or RESOURCE	SOURCE	IDENTIFIER
**Antibodies**		

anti-GAPDH	Elabscience, Houston, TX, USA	Cat#E-AB-20059; RRID:AB_2905551
anti-FLAG	Sigma-Aldrich, St Louis, MO, USA	Cat#F2555; RRID:AB_796202
anti-H2K27me3	Abcam, Cambridge, UK	Cat#ab6147; RRID:AB_449502
anti-E Cadherin	Abcam, Cambridge, UK	Cat#ab40772; RRID:AB_731493
anti-Vimentin	Abcam, Cambridge, UK	Cat#ab92547; RRID:AB_10562134
anti-BMP2	Affinity Biosciences, Jiangsu, China	Cat#AF5163; RRID:AB_2837649
anti-Smad1/5/8	Affinity Biosciences, Jiangsu, China	Cat#AF8313; RRID:AB_2840375
anti-P21	Affinity Biosciences, Jiangsu, China	Cat#AF6290; RRID:AB_2827699
anti-P53	Affinity Biosciences, Jiangsu, China	Cat#AF0879; RRID:AB_2827700
anti-Cyclin D1	Affinity Biosciences, Jiangsu, China	Cat#AF0931; RRID:AB_2835325
anti-CDK4	Affinity Biosciences, Jiangsu, China	Cat#DF6102; RRID:AB_2838070
anti-CDK6	Affinity Biosciences, Jiangsu, China	Cat#DF6448; RRID:AB_2838411
anti-SNRPB	Abcam, Cambridge, UK	Cat#ab155026; RRID:N/A
goat anti-rabbit IgG	Abclonal, Wuhan,China	Cat##AS014; RRID:AB_2769854
goat anti-mouse IgG	Abclonal, Wuhan,China	Cat##AS003; RRID:AB_2769851

**Biological samples**		

human hepatocellular carcinoma (HCC) samples	the First Affiliated Hospital of Anhui Medical University (Hefei, China)	N/A

**Chemicals, peptides, and recombinant proteins**		

DMEM	Invitrogen,CA,USA	Cat#11965092
jetPRIME transfection reagent	PolyPlus-transfection, Illkirch, France	Cat#101000006
RIPA lysis buffer	Beyotime, Shanghai, China	Cat#P0039-100mL
Tris-buffered saline	Servicebio, Wuhan, China	Cat#G0001-2L
TRIzol reagent	Invitrogen, CA, USA	Cat#15596018CN
SuperScript III reverse transcriptase	Life Technologies,Carlsbad, CA, USA	Cat#18080044
SYBR Premix Ex TaqTM II	TaKaRa,Otsu, Shiga, Japan	Cat#640022
CCK-8 detection kit	Beyotime,Shanghai, China	Cat#C0037
Matrigel	BD Biosciences, San Jose, CA, USA	Cat#356234
RNA extraction kit	ESScience,Shanghai, China	Cat#ES-RN001

**Deposited data**		

Raw sequence data	This study	GSE189372

**Experimental models: Cell lines**		

HepG2	College of Basic Medical Sciences, Anhui Medical University, China	N/A
MHCC97-L	College of Basic Medical Sciences, Anhui Medical University, China	N/A
HepG3B	College of Basic Medical Sciences, Anhui Medical University, China	N/A
HCCLM3	College of Basic Medical Sciences, Anhui Medical University, China	N/A
Huh-7	College of Basic Medical Sciences, Anhui Medical University, China	N/A

**Oligonucleotides**		

siSNRPB-1	Generalbiol, Anhui, China	5′-CAAGCCAAAGAACUCCAAA-3′
siSNRPB-1	Generalbiol, Anhui, China	5′-GGACCUCCUCCCAAAGAUA-3′
siEZH2-A	Generalbiol, Anhui, China	5′-AAGAGGUUCAGACGAGCUGAUUU-3′
siEZH2-C	Generalbiol, Anhui, China	5′-CTAGGGAGGTGGAAGATGAAACT-3′
siEZH2-C	Generalbiol, Anhui, China	5′-AAAGGGTCAAAACCGCTTTCCGG-3′

**Recombinant DNA**		

Flag-tagged EZH2-A	Fubio, Suzhou, China	https://fubio.cn/
Flag-tagged EZH2-B	Fubio, Suzhou, China	https://fubio.cn/
Flag-tagged EZH2-C	Fubio, Suzhou, China	https://fubio.cn/

**Software and algorithms**		

JBrowse	https://jbrowse.org/jb2/	N/A
GEPIA2	http://gepia2.cancer-pku.cn/	N/A

### Experimental model and study participant details

#### The cell lines

HepG2, MHCC97-L, HepG3B, HCCLM3, and Huh-7 were preserved in the laboratory. The cells were cultured in Dulbecco’s Modified Eagle Medium (DMEM) medium supplemented with 5% fetal bovine serum (FBS) and 1% antibiotics in a 37°C incubator with 5% CO2. All cell lines were authenticated by short tandem repeat PCR profiling. All cell lines were routinely tested negative for mycoplasma contamination with a mycoplasma assay kit (Yeasen, Shanghai, China).

#### HCC samples

This study utilized 28 HCC samples and the normal tissues adjacent to the tumor (22 male and 6 female) were obtained from the First Affiliated Hospital of Anhui Medical University (Hefei, China, ethical approval number: No. 20200839). The samples collected from the hospitals were divided into cancerous and paraneoplastic tissues and stored at −80°C. The handling of patient specimens in this study adhered to the regulations set by the National Health Care Commission and the principles outlined in the Declaration of Helsinki, and informed consent has been obtained from all subjects. Table S1 provides a summary of the patient sample information (Including information on the sex, race and age of all study participants, as well as sample size and how subjects/samples were assigned to experimental groups). No male/female differences were expected in this experiment, so we collected HCC samples regardless of their sex.

#### Sequencing

The cDNAs of EZH2 splice variants were amplified using specifically designed primers listed in Table S2. Sanger sequencing was performed by Qingke Biotechnology Co., Ltd, and the sequences were aligned by Chromas (Technelysium Pty Ltd., Tewantin, Australia). Next generation RNA sequencing was performed on an Illumina platform with 150 bp paired-end reads (Novogene Bioinformatic Technology Co., Ltd). Raw reads were filtered by FastQC v0.11.8 and TrimGalore v0.6.2, and the clean reads were then mapped to the human genome (GRCh38) using STAR v2.6.1a.^52^

#### Software forecasting

The Ensembl gene browser database was used to query specific EZH2 variants. Sequences of cDNAs and amino acids were also downloaded from Ensembl. The levels of EZH2 splice variants in human tissues were obtained from the Genotype-Tissue Expression (GTEx) project and analyzed using GTExPortal (https://www.gtexportal.org/home/). The sequencing data of HepG2 cells from the PacBio platform in ENCODE (ENCSR834DQL, https://www.encodeproject.org/fifiles/ENCFF470UHX/) (accessed on 13 July 2020) were downloaded, and the transcripts were visualized using JBrowse.^53^^,^^54^ Isoform levels and patient survival analyses were performed using GEPIA2.^55^

All plasmids were designed and synthesized by Fubio (Suzhou, China), and detailed instructions and plasmid tests have been uploaded to Mendeley data. All siRNAs are synthesized by Generalbiol (Anhui, China).

### Method details

#### Cell transfection

The pcDNA3.1 vector was used to generate Flag-tagged EZH2-A (WT) and its variants (-B, -C). The HCC cell lines were transiently transfected using the jetPRIME transfection reagent (PolyPlus-transfection, Illkirch, France). Transfection step: 1000 ng of plasmid was added to 200ul jet prime buffer, vortexed for 10s and dumped in a small centrifuge. Add 4μL JetPRIME reagent to the above tube, vortex for 10s, spin at reduced speed. Incubate at RT for 10 to 15 min. Add the transfection mixture dropwise to the cells in serum-containing medium. Gently rock the six-well plate back and forth, rocking from side to side, and incubate at 37°C. Replace transfection medium with cell growth medium 24 h after transfection. And same steps for siRNA transfection。The concentration was measured and reverse transcription was performed to obtain cDNA, using SuperScript III reverse transcriptase (Life Technologies, Carlsbad, CA, USA).

#### Western blotting

RNA and protein were collected 48–72 h after transfection. The RNA extraction procedure is described in the instructions of the RNA extraction kit from ESScience (ES-RN001,ESScience, Shanghai, China. http://www.esunbio.com/search-1--/u0072/u006e/u0061.html). Total proteins were extracted with RIPA lysis buffer (Beyotime, Shanghai, China). Proteins were separated using 400 μL of SDS-PAGE gels and then transferred to polyvinylidene fluoride (PVDF) membranes. The membrane was incubated in Tris-buffered saline (TBS) at room temperature for 2 h using 5% skim milk. Washing 3 times with TBST (2 L TBS+1 mL Tween 20) and then incubated overnight at 4°C with primary antibodies. Primary antibodies were contacted with horseradish peroxidase (HRP)-conjugated goat anti-rabbit IgG (1:8000) or goat anti-mouse IgG (1:8000) for 2 h and then washed 3 times with TBST. The detection of the secondary antibodies was achieved using ECL Prime Western blotting Detection Reagents.

#### Quantitative real-time PCR

Total RNAs were collected from hepatocellular carcinoma (HCC) patients and HCC cells using TRIzol reagent (Invitrogen, CA, USA) as previous report.^11^ Harvest cells 1-5×107, transfer to 1.5mL centrifuge tube, add 1mL Trizol, mix well, and let stand at room temperature for 5min.Tissue: Take 50-100mg of tissue (fresh or tissue stored at −70°C and liquid nitrogen can be used) into a 1.5mL centrifuge tube, add 1mL Trizol, homogenise well, and let stand at room temperature for 5min.Add 0.2mL of chloroform, shake for 15s, and let stand for 2min. Add 0.2mL of chloroform, shake for 15s, and leave for 2min. Centrifuge at 4°C, 12000 g × 15 min, and take the supernatant. Add 0.5mL isopropanol, mix the liquid in the tube gently, let stand at room temperature for 10min centrifuge at 4°C, 12000 g × 10 min, discard the supernatant. Add 1mL 75% ethanol, gently wash the precipitate. 4°C, 7500 g × 5 min, discard the supernatant. Let dry, add appropriate amount of DEPC H2O to dissolve (65°C to promote dissolution for 10-15min). The collected RNA was stored at −80°C for later use. We used SuperScript III reverse transcriptase (Life Technologies, Carlsbad, CA, USA) for the reverse transcription. For quantitative PCR analysis, Applied Biosystems StepOnePlus Real-Time PCR System and SYBR Premix Ex TaqTM II (TaKaRa, Otsu, Shiga, Japan) was used. The obtained data were subsequently analyzed using the 2-ΔΔCq method^56^ and normalized to glyceraldehyde 3-phosphate dehydrogenase (GAPDH).

#### Immunofluorescence staining

HCC cell lines were transfected with plasmids (including empty plasmid as negative control, EZH2-A, EZH2-B, EZH2-C) for immunofluorescence staining, and subsequently cultured on 13-mm coverslips for 48 h. The cells were fixed with a 4% formaldehyde dilution for 30 min at room temperature after being rinsed with PBS. Afterward, the cells underwent three rounds of washing with PBS for 5 min each. Subsequently, they were permeabilized using a solution containing 0.5% Triton X-100 in PBS for a duration of 20 min. Following that, the cells were blocked using 10% normal goat serum for 30 min at a temperature of 37°C. The cells were then subjected to an overnight incubation at a temperature of 4°C, with the presence of the anti-FLAG antibody (1:200). After that, the cells were rinsed three times with PBS and then exposed to the respective secondary antibodies (goat anti-Mouse IgG, Alexa Fluor 594, AB0152, Abways) for 1 h at room temperature. Cells were then incubated with DAPI and mounted with mounting medium. Using established and constant settings, cells were imaged by confocal microscopy (LSM880, Carl Zeiss MicroImaging, Inc.).

#### Cell growth and proliferation assays

To assess the growth capacity of cells, we employed the CCK-8 detection kit (Beyotime, Shanghai, China) and HCC cell lines were transfected with plasmids (including empty plasmid as negative control, EZH2-A, EZH2-B, EZH2-C) for cell growth and proliferation assays, Prepare cell suspension and count, inoculate cell suspension in 96-well plate, about 100μL per well, the same sample can do 5 replicates, put the plate into the incubator for a period of time pre-culture (37°C, 5% CO2), directly configure the medium containing 10% CCK-8, add in the form of a change of liquid, put the plate into the incubator and incubate for 2h, the zymography to determine the absorbance at 450nm (OD) on 4 consecutive days (0, 24, 48 and 72 h).

#### Cell migration and invasion assays

The wound healing assay was performed as previous report.^11^ The cells were grown in cell culture plates at specified densities. Once the cells reached 80%–90% coverage, a scratching tool was used to create a wound in the center of the six-well plate. The culture medium was then replaced. Pictures of the scratched area were taken at specified times. The size of the wound was measured using ImageJ software. To evaluate the invasive ability of cells, a transwell system (Corning, NY, USA) was employed. Specifically, the upper chamber of the transwell system was filled with Matrigel (BD Biosciences, San Jose, CA, USA) and 5×10^4^ cells in serum-free DMEM. Simultaneously, the lower chamber was supplied with DMEM containing 20% FBS. After 24 h, the cells that had migrated beneath the membrane surface were fixed with 4% paraformaldehyde for 30 min and then stained with 0.5% crystal violet (Servicebio, Wuhan, China) for 10 min. The transwell membranes, which contained the stained cells, were captured digitally and subsequently counted using the ImageJ software.

### Quantification &statistical analysis

All statistical analyses were conducted using GraphPad Prism software. Differences between groups were evaluated by presenting the results as the mean ± standard deviation. Student’s t-test was utilized to evaluate statistical significance, and *p*-values less than 0.05 indicate statistical significance. ∗ indicates a statistically significant difference compared with the control group, *p* < 0.5, ∗∗indicates a statistically significant difference compared with the control group, *p* < 0.01, ∗∗∗ indicates a statistical difference compared with the control group, *p* < 0.001, and ‘∗∗∗∗’ indicates a statistical difference compared with the control group, *p* < 0 0.0001.
