# Supplementary material for: Alternative splicing of EZH2 regulated by SNRPB mediates hepatocellular carcinoma progression via BMP2 signaling pathway
Source: iScience. 2024 Dec 18;28(1):111626. doi: 10.1016/j.isci.2024.111626 (PMC11754826; doi:10.1016/j.isci.2024.111626)
Supplement: Document S1. Figures S1–S9 and Tables S1 and S2 [file mmc1.pdf]

iScience, Volume *28*

Supplemental information

Alternative splicing of EZH2 regulated by SNRPB mediates hepatocellular carcinoma progression via BMP2 signaling pathway

Xingyu Wang, Weiyi Liu, Chunai Zhan, Yuanyuan Zhang, Xinyu Li, Yaoyun Wang, Mengfei Sheng, Madiha Maqsood, Hang Shen, Anmin Liang, and Wei Shao

Figure S1 Alternatively Spliced Isoforms of EZH2 in Human Tissues

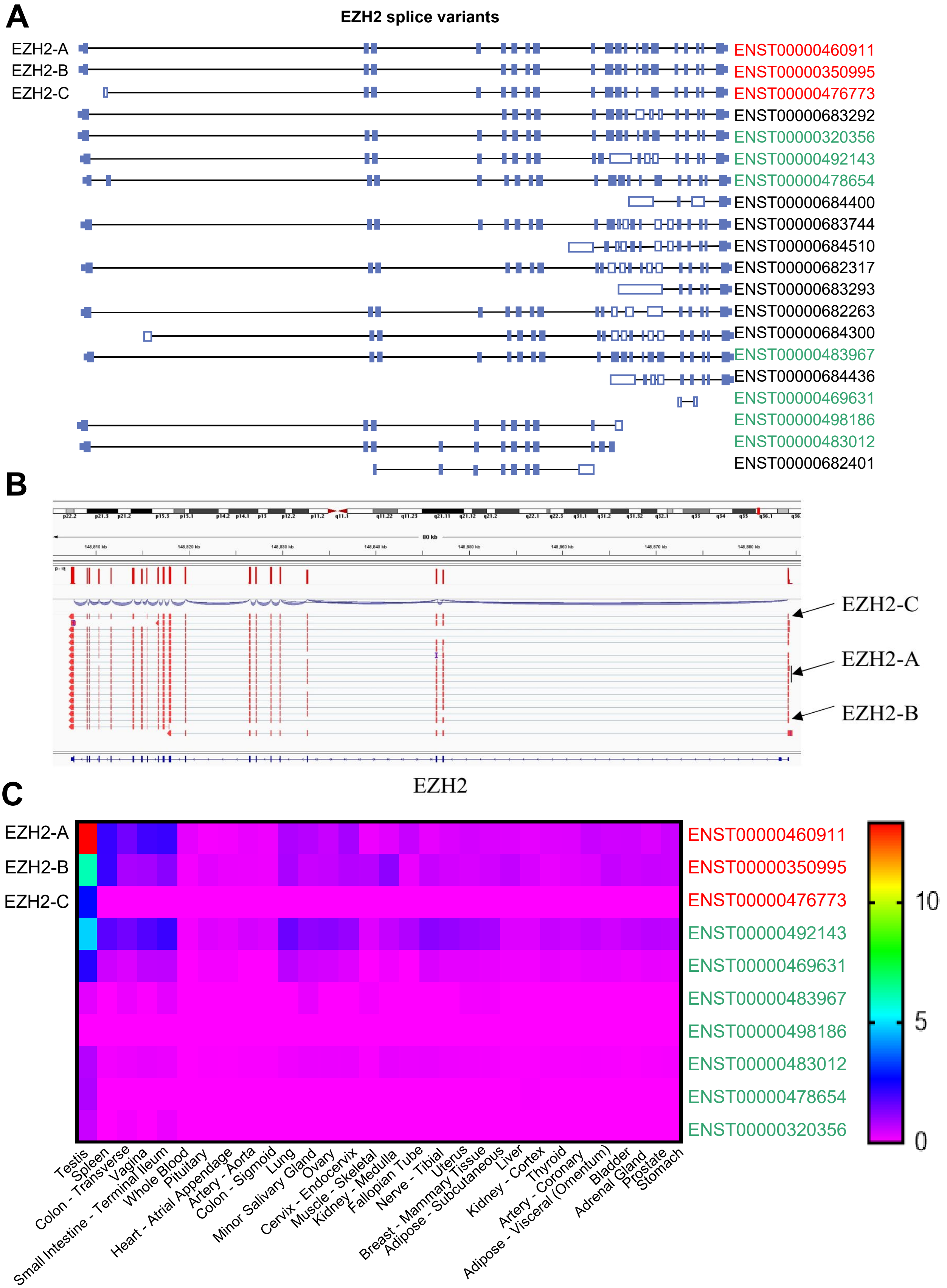

Figure S1. Alternatively spliced isoforms of EZH2 in human tissues, related to Figure 1.

(A) List of all detectable mRNA isoforms of EZH2. The original data were downloaded from the Ensembl gene browser database. Three isoforms, EZH2-A, -B, and -C, were further studied.
(B) The full sequence of the EZH2-A, B and C variant were analyzed using third-generation sequencing data of HepG2 cells. EZH2-A, B and C variants were visualized by the integrative genomics viewer (IGV).
(C) Heatmap of EZH2 variants mRNA expression levels in different human tissues. The original data were obtained from the Genotype-Tissue Expression Project (GTExPortal).

Figure S2 Primary Sequences of the Proteins Encoded by the Three EZH2 Isoforms

A

```
EZH2-A    MGQTGKKSEKGPVCWRKRVKSEYMRLRQLKRFRRADEVKSMFSSNRQKILERTEILNQEWKQRRIQPVHILTSVSSL
EZH2-B    MGQTGKKSEKGPVCWRKRVKSEYMRLRQLKRFRRADEVKSMFSSNRQKILERTEILNQEWKQRRIQPVHILTSVSSL
EZH2-C    MGQTGKKSEKGPVCWRKRVKSEYMRLRQLKRFRRADEVKSMFSSNRQKILERTEILNQEWKQRRIQPVHILTS----

EZH2-A    RGTRECSVTSDLDFPTQVIPLKTLNAVASVPIMYSWSPLQQNFMVEDETVLHNIPYMGDEVLDQDGTFIEELIKNYD
EZH2-B    RGTRE--------------------------------------VEDETVLHNIPYMGDEVLDQDGTFIEELIKNYD
EZH2-C    -----CSVTSDLDFPTQVIPLKTLNAVASVPIMYSWSPLQQNFMVEDETVLHNIPYMGDEVLDQDGTFIEELIKNYD

EZH2-A    GKVHGDRECGFINDEIFVELVNALGQYNDDDDDDDGDDPEEREEKQKDLEDHRDDKESRPPRKFPSDKIFEAISSMF
EZH2-B    GKVHGDRECGFINDEIFVELVNALGQYNDDDDDDDGDDPEEREEKQKDLEDHRDDKESRPPRKFPSDKIFEAISSMF
EZH2-C    GKVHGDRECGFINDEIFVELVNALGQYNDDDDDDDGDDPEEREEKQKDLEDHRDDKESRPPRKFPSDKIFEAISSMF

EZH2-A    PDKGTAEELKEKYKELTEQQLPGALPPECTPNIDGPNAKSVQREQSLHSFHTLFCRRCFKYDCFLHPFHATPNTYKR
EZH2-B    PDKGTAEELKEKYKELTEQQLPGALPPECTPNIDGPNAKSVQREQSLHSFHTLFCRRCFKYDCFLHPFHATPNTYKR
EZH2-C    PDKGTAEELKEKYKELTEQQLPGALPPECTPNIDGPNAKSVQREQSLHSFHTLFCRRCFKYDCFLHPFHATPNTYKR

EZH2-A    KNTETALDNKPCGPQCYQHLEGAKEFAAALTAERIKTPPKRPGGRRRGRLPNNSSRPSTPTINVLESKDTDSDREAG
EZH2-B    KNTETALDNKPCGPQCYQHLEGAKEFAAALTAERIKTPPKRPGGRRRGRLPNNSSRPSTPTINVLESKDTDSDREAG
EZH2-C    KNTETALDNKPCGPQCYQHLEGAKEFAAALTAERIKTPPKRPGGRRRGRLPNNSSRPSTPTINVLESKDTDSDREAG

EZH2-A    TETGGENNDKEEEEKKDETSSSSEANSRCQTPIKMKPNIEPPENVEWSGAEASMFRVLIGTYYDNFCAIARLIGTKT
EZH2-B    TETGGENNDKEEEEKKDETSSSSEANSRCQTPIKMKPNIEPPENVEWSGAEASMFRVLIGTYYDNFCAIARLIGTKT
EZH2-C    TETGGENNDKEEEEKKDETSSSSEANSRCQTPIKMKPNIEPPENVEWSGAEASMFRVLIGTYYDNFCAIARLIGTKT

EZH2-A    CRQVYEFRVKESSIIAPAPAEDVDTPPRKKKRKHRLWAAHCRKIQLKKDGSSNHVYNYQPCDHPRQPCDSSCPCVIA
EZH2-B    CRQVYEFRVKESSIIAPAPAEDVDTPPRKKKRKHRLWAAHCRKIQLKKDGSSNHVYNYQPCDHPRQPCDSSCPCVIA
EZH2-C    CRQVYEFRVKESSIIAPAPAEDVDTPPRKKKRKHRLWAAHCRKIQLKKG----------------------------

EZH2-A    QNFCEKFCQCSSECQNRFPGCRCKAQCNTKQCPCYLAVRECDPDLCLTCGAADHWDSKNVSCKNCSIQRGSKKHLLL
EZH2-B    QNFCEKFCQCSSECQNRFPGCRCKAQCNTKQCPCYLAVRECDPDLCLTCGAADHWDSKNVSCKNCSIQRGSKKHLLL
EZH2-C    --------------QNRFPGCRCKAQCNTKQCPCYLAVRECDPDLCLTCGAADHWDSKNVSCKNCSIQRGSKKHLLL

EZH2-A    APSDVAGWGIFIKDPVQKNEFISEYCGEIISQDEADRRGKVYDKYMCSFLFNLNNDFVVDATRKGNKIRFANHSVNP
EZH2-B    APSDVAGWGIFIKDPVQKNEFISEYCGEIISQDEADRRGKVYDKYMCSFLFNLNNDFVVDATRKGNKIRFANHSVNP
EZH2-C    APSDVAGWGIFIKDPVQKNEFISEYCGEIISQDEADRRGKVYDKYMCSFLFNLNNDFVVDATRKGNKIRFANHSVNP

EZH2-A    NCYAKVMMVNGDHRIGIFAKRAIQTGEELFFDYRYSQADALKYVGIEREMEIP
EZH2-B    NCYAKVMMVNGDHRIGIFAKRAIQTGEELFFDYRYSQADALKYVGIEREMEIP
EZH2-C    NCYAKVMMVNGDHRIGIFAKRAIQTGEELFFDYRYSQADALKYVGIEREMEIP
```

B

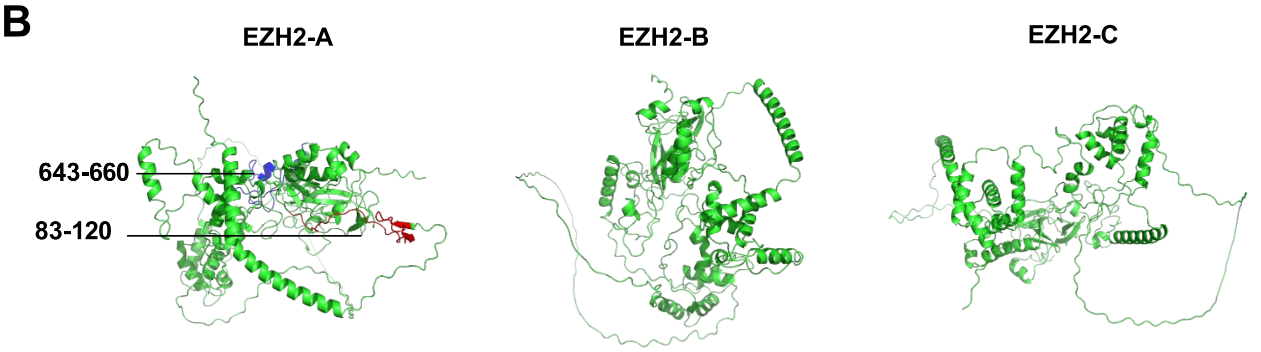

Figure S2 Amino acid sequences of the proteins encoded by the three EZH2 isoforms, related to Figure 1.

(A) Sequence alignment of EZH2 isoforms shows a divergence in the C-terminal region. Red letters indicate the different amino acids.

(B) Prediction of protein structures of different EZH2 splice variants by AlphaFold.

Figure S3 Validation and analysis of EZH2 splicing variants

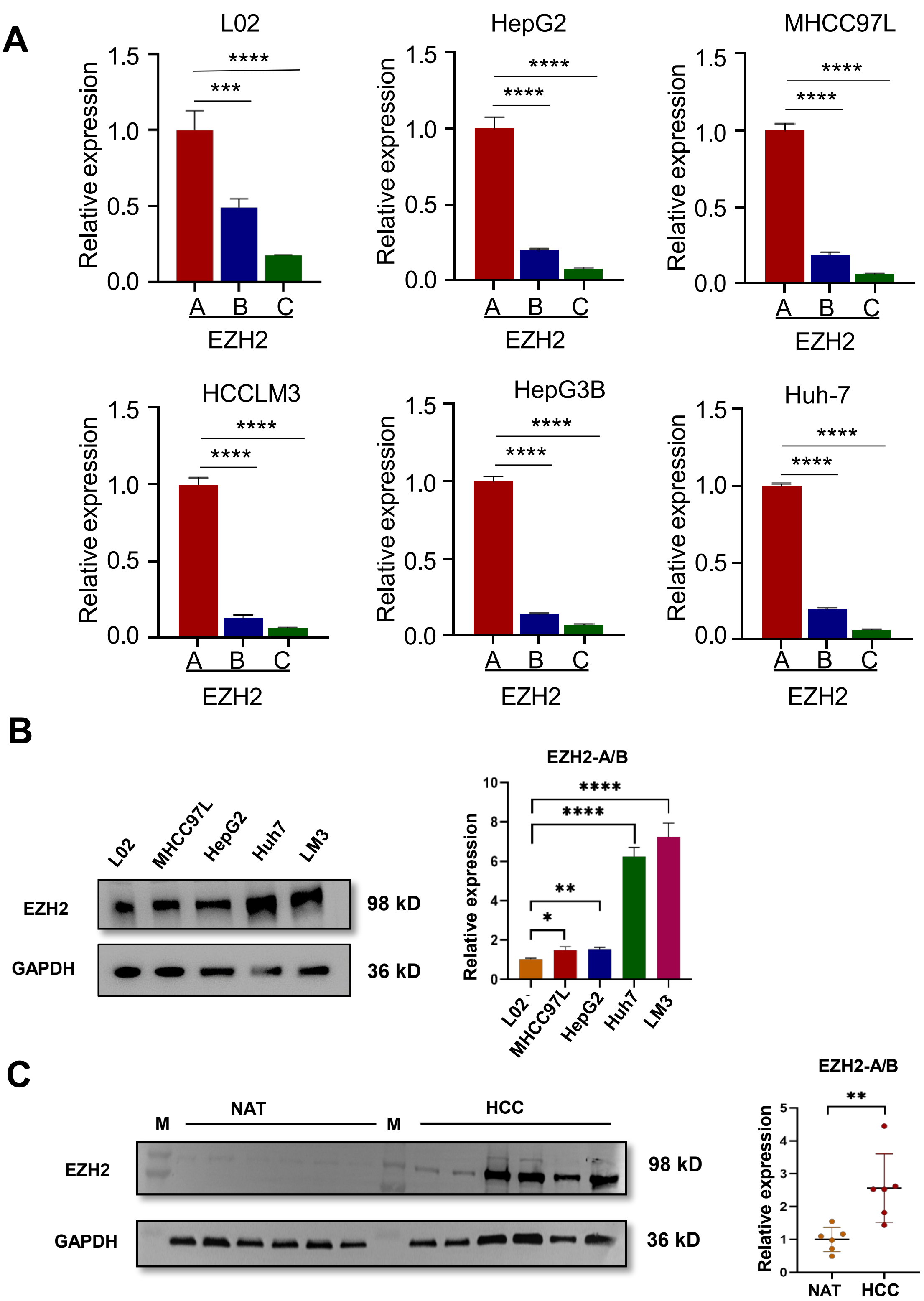

Figure S3 Validation and analysis of EZH2 splicing variants, related to Figure 2.
(A) RT-qPCR analysis of EZH2-A, -B, and -C in normal liver cell line L02 and five hepatocellular carcinoma cell lines (HepG2, MHCC97L, HCCLM3, HepG3B, Huh-7).
(B and C) Western blots were used to detect the proteins expression of EZH2 variants in normal liver cell line L02 and HCC cell lines (B) and human samples (C). The molecular weights of EZH2-A and EZH2-B are too close to be separated by Western blotting, the bands represent EZH2-A and B. NAT: normal tissue adjacent to the tumor. M: marker.
The data are presented as means ± SD. *$P < 0.05$, **$P < 0.01$, ***$P < 0.001$, ****$P < 0.0001$ (t test).

Figure S4 mRNA levels of EZH2-A and EZH2-B in different cancers

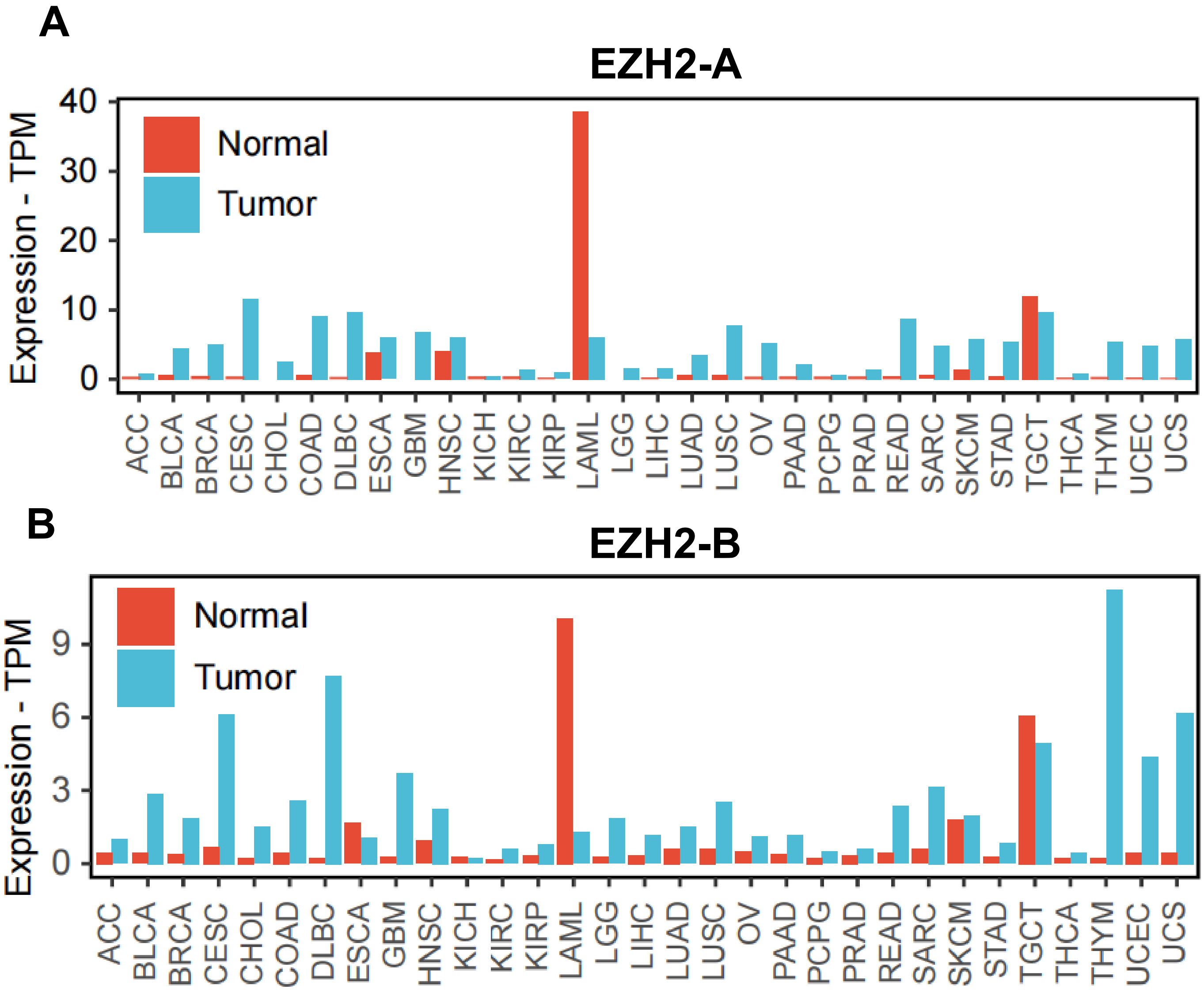

Figure S4 mRNA Levels of three EZH2 isoforms in different cancers, related to Figure 2. Levels of EZH2-A (A), B (B) mRNAs in normal and tumor tissues from different cancers were analyzed using data from the GEPIA2 database.

Figure S5 Correlation between the Levels of the EZH2 splice Variant and Patient Survival in Multiple Cancers

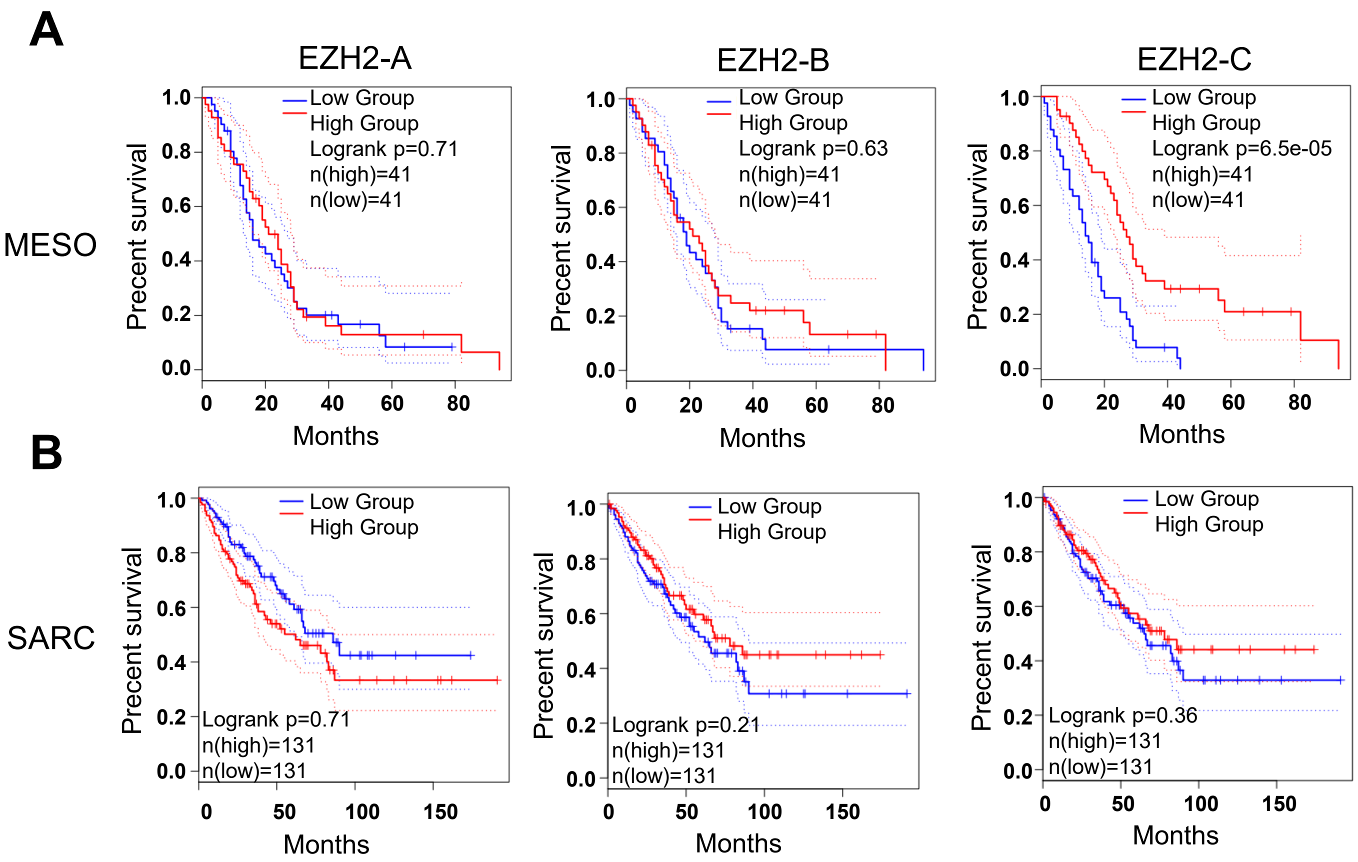

Figure S5 Correlation between the levels of the EZH2-C variant and patient survival in multiple cancers, related to Figure 2.
Patients with higher EZH2-C expression in MESO (A) and SARC (B) have longer median overall survival lengths.

Figure S6 Expression of Flag-tagged EZH2 variants.

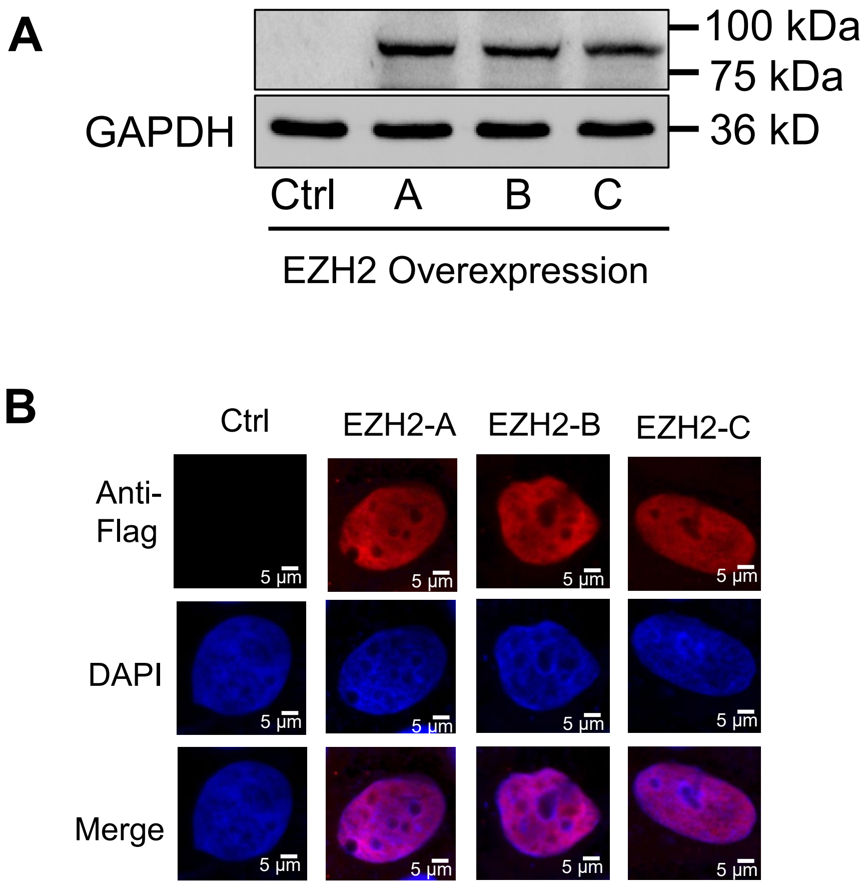

Figure S6 Expression of Flag-tagged EZH2 variants, related to Figure 3.

(A) Western blot analysis of HepG2 cells expressing Flag-tagged EZH2 variants using the anti-FLAG antibody.

(B) Immunofluorescence analysis of ectopically expressed Flag-tagged EZH2 and its isoforms in HepG2. Scale bar: 25 μm

Figure S7 Silencing of EZH2 variants regulates HCC cell proliferation.

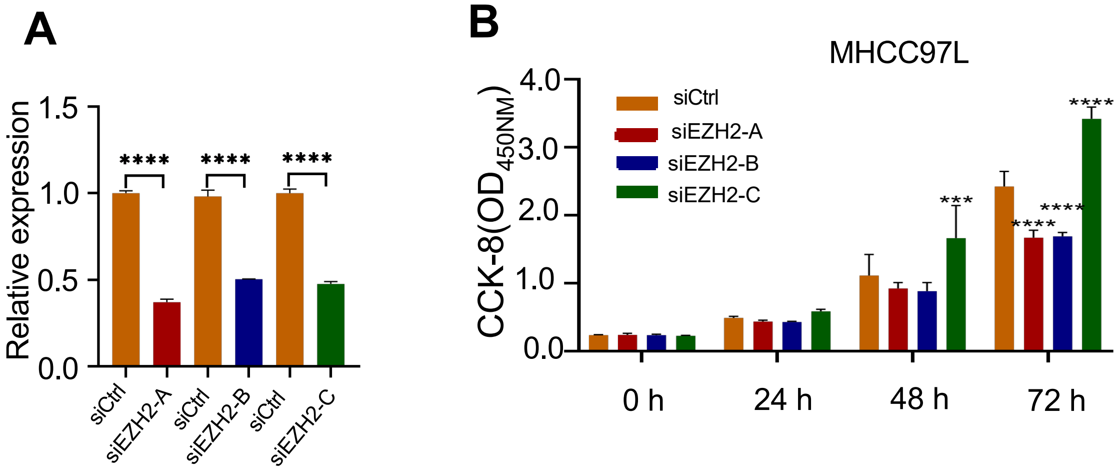

Figure S7 Silencing of EZH2 variants regulates HCC cell proliferation, related to Figure 3.
(A) siRNA knock down efficiency of EZH2 variants.
(B) Splice variants specific siRNA of EZH2-A, -B or -C were transfected into MHCC97L cells. The cell proliferative activity was assessed by CCK-8 assay.
The data are presented as means ± SD. *P < 0.05, **P < 0.01, ***P < 0.001, ****P < 0.0001 (t test).)

Figure S8 EZH2-C Inhibits the Migration of Cells

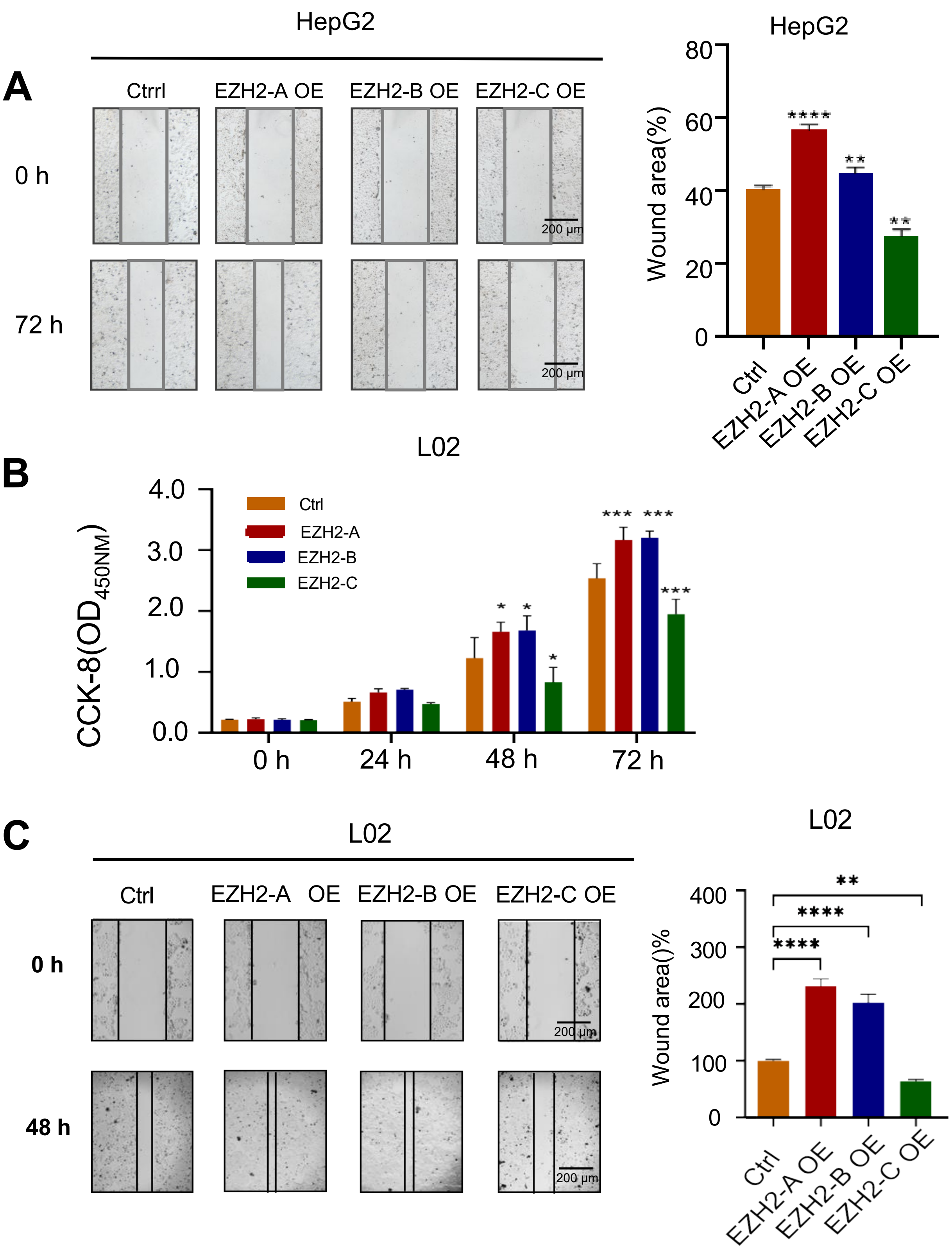

Figure S8 EZH2-C inhibits the migration of HepG2 and LO2 cells, related to Figure 3.
(A) Migration was determined by scratch experiments. Expression of EZH2-A, -B or -C plasmids were transfected into HepG2 cells. Representative images are shown at 0 h and 48 h after transfections. Scale bar, 200 μm
(B and C) Altered proliferative capacities (B) and migratory (C) of normal liver cell line L02 after transfection with EZH2-A, B and C plasmids. Scale bar, 200 μm
The data are presented as means ± SD. *$P < 0.05$, **$P < 0.01$, ***$P < 0.001$, ****$P < 0.0001$ (t test).

Figure S9 Silencing of EZH2 variants regulates the expression of EZH2 target genes.

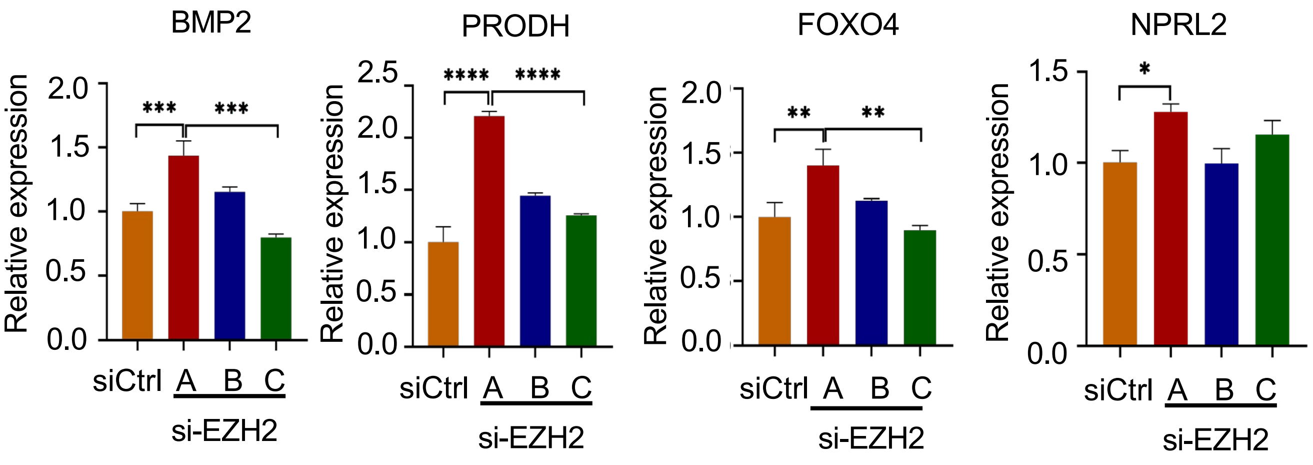

Figure S9 Silencing of EZH2 variants regulates the expression of EZH2 target genes, related to Figure 5.

Splice variants specific siRNA of EZH2-A, -B or -C were transfected into MHCC97L cells. Expression levels of BMP2, PRODH, FOXO4, NPRL2 were detected by RT-qPCR.

The data are presented as means ± SD. *P < 0.05, **P < 0.01, ***P < 0.001, ****P < 0.0001 (t test).

Table S1: Clinical characteristics of patients

No. of Patients	Age	Sex	Race	Liver cirrhosis Yes=1 No=2	Solitary=2 Multiple=1	Lymph node metastasis Yes=1 No=2	Tumor size (cm)	TNM stage	Stage	Hemoglobin (g/dL)	WBC (10^9/L)	Platelet (10^9/L)	ALB (g/L)	Blood urea nitrogen (mmol/L)	Creatinine (μmol/L)	Total bilirubin (μmol/L)	ALT (μ/L)	AST (μ/L)	Used in this study
1	57	male	Chinese	1	1	2	3.5	T1bN0M0	IB	140	3.84	146	36.3	7.7	9.4	21.3	252	283	RT-qPCR
2	72	male	Chinese	1	1	2	2.5	T1bN0M0	IB	141	6.5	255	37.7	5.7	88	16.9	17	18	RT-qPCR
3	59	male	Chinese	1	1	2	11.0	T1bN0M0	IB	140	6.25	192	23.9	10.04	112	15	78	79	RT-qPCR
4	50	female	Chinese	2	1	2	4.5	T1bN0M0	IB	141	7.96	213	39.1	3.8	4.25	19	19	19	RT-qPCR
5	60	male	Chinese	1	1	2	11.0	T1bN0M0	IB	99	/	/	28.7	/	/	/	/	/	RT-qPCR
6	56	male	Chinese	2	1	2	6.5	T1bN0M0	IB	135	4.33	103	45.5	5.6	68	23.4	26	25	RT-qPCR
7	41	female	Chinese	1	1	2	3.5	T1bN0M0	IB	140	/	/	/	/	/	/	/	/	RT-qPCR
8	47	male	Chinese	1	1	2	6.0	T1bN0M0	IB	134	8.25	124	42.7	4.64	106	28.9	42	79	RT-qPCR
9	36	female	Chinese	2	1	2	4.0	T1bN0M0	IB	87	4.33	209	41.8	5.07	66	6.5	13	18	RT-qPCR
10	49	male	Chinese	2	1	2	9.0	T4N0M0	IIIB	125	9.16	92	33.9	5.52	50	25.2	38	56	RT-qPCR
11	41	male	Chinese	2	2	2	13.0	T3N0M0	IIIA	144	6.46	169	36.2	6.06	68	40.2	19	44	RT-qPCR
12	46	male	Chinese	1	2	2	11.0	T4N0M0	IIIB	155	6.71	198	40	3.25	64	9	33	48	RT-qPCR
13	74	male	Chinese	1	1	2	15.0	T4N0M0	IIIB	122	6.06	155	27.9	3.62	81	14.9	130	180	RT-qPCR
14	57	male	Chinese	1	1	2	13.0	T4N0M0	IIIB	119	4.22	55	38.5	9.19	97	7.7	47	50	RT-qPCR
15	41	male	Chinese	1	2	2	8.0	T4N0M0	IIIB	118	15.78	193	36.3	4.42	91	10	36	45	RT-qPCR
16	62	female	Chinese	1	1	2	3.0	T4N0M0	IIIB	134	3.62	100	40.3	4.49	56	16.6	33	32	RT-qPCR
17	73	male	Chinese	1	2	2	8.0	T4N0M0	IIIB	146	5.35	106	33.8	5.77	70	18	30	42	RT-qPCR
18	69	male	Chinese	2	1	2	4.7	T4N0M0	IIIB	93	7.65	190	34.8	5.3	86	23	37	37	RT-qPCR
19	67	female	Chinese	1	2	2	4.5	/	II	118	3.94	83	39.9	6.63	73	17.6	26	33	RT-qPCR/Western Blot
20	47	male	Chinese	2	2	2	11.5	/	III	159	5.67	126	41.8	5.7	79	17.3	154	85	RT-qPCR/Western Blot
21	59	female	Chinese	2	2	1	9.0	/	/	104	9.75	339	40	4.02	65	7.3	17	18	RT-qPCR/Western Blot
22	61	male	Chinese	1	1	2	2.6	/	II-III	188	5.69	133	46.6	4.72	99	15.3	44	29	RT-qPCR
23	74	male	Chinese	1	2	2	4.1	/	II	146	5.59	107	48	6.29	77	14.1	21	30	RT-qPCR
24	61	male	Chinese	1	2	2	6.5	/	III	127	8.42	198	44.3	4.93	88	13.8	52	22	RT-qPCR
25	74	male	Chinese	2	2	2	4.2	/	III	154	6.47	181	48.1	4.54	94	11.3	15	22	RT-qPCR
26	69	male	Chinese	1	2	2	3.6	/	II-III	136	2.49	86	39	5.75	78	10.9	26	47	RT-qPCR
27	72	male	Chinese	2	2	2	7.8	/	II	161	5.56	247	46.6	4.55	85	17.1	32	31	RT-qPCR
28	49	male	Chinese	1	2	2	2.2	/	/	177	5.27	113	49.8	6.32	73	6.7	37	30	RT-qPCR

Table S2 List of primers used in this study

Primers for qRT-PCR		
Gene	Human/Mouse	sequence(5'-3')
EZH2-A	Human	F: GACTTCTGTGAGCTCATTGC
EZH2-A	Human	R: TGGGTACTGAAGCAACTGCA
EZH2-B	Human	F: CTAGGGAGGTGGAAGATGAA
EZH2-B	Human	R: TAAACCCACATTCTCTATCC
EZH2-C	Human	F: AAAGGGTCAAAACCGCTTTC
EZH2-C	Human	R: TTACTGTCCCAATGGTCAGC
PRODH	Human	F: ACGAATAAGCGGGACAAGCA
PRODH	Human	R: CCGTCATCGCTGACTCTACC
FOXO4	Human	F: ACGAGTGGATGGTCCGTACTGT
FOXO4	Human	R: CCTTGATGAACTTGCTGTGCAGG
NPRL2	Human	F: GGACCTCACTACACAACAAATCCTG
NPRL2	Human	R: GTCACAACGCCGTAGTACAGCA
BMP2	Human	F: TGTATCGCAGGCACTCAGGTCA
BMP2	Human	R: CCACTCGTTTCTGGTAGTTCTTC
GAPDH	Human	F: TCAAGTGGGGCGATGCTGGC
GAPDH	Human	R: TGGGGGCATCAGCAGAGGGG
**Primers for PCR**		
EZH2-A/B	Human	F: GACTTCTGTGAGCTCATTGC
EZH2-A/B	Human	R: GACCAAGGGCATTCACCAAC
EZH2-A/C	Human	F: GACAATTTCTGTGCCATTGC
EZH2-A/C	Human	R: ACTCTCGGACAGCCAGGTAG
**Primers for SNRPB-siRNA**		
siSNRPB-1	Human	5′-CAAGCCAAAGAACUCCAAA-3′,
siSNRPB-2	Human	5′-GGACCUCCUCCCAAAGAUA-3′
siEZH2-A	Human	5'-AAGAGGUUCAGACGAGCUGAUUU-3'
siEZH2-B	Human	5'-CTAGGGAGGTGGAAGATGAAACT-3'
siEZH2-C	Human	5'-AAAGGGTCAAAACCGCTTTCCGG-3'
